# The Serotonergic Central Nervous System of the *Drosophila* Larva: Anatomy and Behavioral Function

**DOI:** 10.1371/journal.pone.0047518

**Published:** 2012-10-17

**Authors:** Annina Huser, Astrid Rohwedder, Anthi A. Apostolopoulou, Annekathrin Widmann, Johanna E. Pfitzenmaier, Elena M. Maiolo, Mareike Selcho, Dennis Pauls, Alina von Essen, Tripti Gupta, Simon G. Sprecher, Serge Birman, Thomas Riemensperger, Reinhard F. Stocker, Andreas S. Thum

**Affiliations:** 1 Department of Biology, University of Fribourg, Fribourg, Switzerland; 2 Neurobiology and Genetics, Theodor-Boveri Institute, Biocenter, University of Würzburg, Würzburg, Germany; 3 Genetic and Physiopathology of Neurotransmission, ESPCI Neurobiology Unit, Centre National de la Recherche Scientifique, Paris, France; 4 Molecular Neurobiology of Behavior, Johann-Friedrich-Blumenbach-Institute for Zoology and Anthropology, Georg-August-University of Goettingen, Goettingen, Germany; 5 Department of Biology, University of Konstanz, Konstanz, Germany; University of Missouri, United States of America

## Abstract

The *Drosophila* larva has turned into a particularly simple model system for studying the neuronal basis of innate behaviors and higher brain functions. Neuronal networks involved in olfaction, gustation, vision and learning and memory have been described during the last decade, often up to the single-cell level. Thus, most of these sensory networks are substantially defined, from the sensory level up to third-order neurons. This is especially true for the olfactory system of the larva. Given the wealth of genetic tools in *Drosophila* it is now possible to address the question how modulatory systems interfere with sensory systems and affect learning and memory. Here we focus on the serotonergic system that was shown to be involved in mammalian and insect sensory perception as well as learning and memory. Larval studies suggested that the serotonergic system is involved in the modulation of olfaction, feeding, vision and heart rate regulation. In a dual anatomical and behavioral approach we describe the basic anatomy of the larval serotonergic system, down to the single-cell level. In parallel, by expressing apoptosis-inducing genes during embryonic and larval development, we ablate most of the serotonergic neurons within the larval central nervous system. When testing these animals for naïve odor, sugar, salt and light perception, no profound phenotype was detectable; even appetitive and aversive learning was normal. Our results provide the first comprehensive description of the neuronal network of the larval serotonergic system. Moreover, they suggest that serotonin *per se* is not necessary for any of the behaviors tested. However, our data do not exclude that this system may modulate or fine-tune a wide set of behaviors, similar to its reported function in other insect species or in mammals. Based on our observations and the availability of a wide variety of genetic tools, this issue can now be addressed.

## Introduction

The classical genetic model system, the fruit fly *Drosophila melanogaster*, shares many of the crucial organizational features of the mammalian central nervous system, yet comprises 1,000 to 10,000 times less neurons [1], [2]. Cell numbers are even more reduced in *Drosophila* larvae, which seem to include no more than 3,000 functional neurons [3]–[6]. Despite this drastic reduction, larvae still display a considerable behavioral repertoire ranging from simple naïve responses such as chemotaxis or phototaxis to higher brain functions like learning and memory [7]–[15]. Thus, many recent studies demonstrate the great potential of *Drosophila* larvae for studying the neuronal basis of behavior [11], [16]–[23].

Current assays for measuring naïve gustatory, olfactory and visual preferences in *Drosophila* larvae are simple choice tests performed on agarose filled Petri dishes [24]. Petri dish assays can also be used to study classical olfactory conditioning. Presenting an odor (the conditioned stimulus [CS]) simultaneously with an aversive unconditioned stimulus (US) may induce experience-dependent avoidance of the CS. Conversely, if the same CS is paired with an appetitive US, animals can be trained to develop a preference for the CS [25]. Thus, depending on previous experience, the same odor can trigger either avoidance or attraction [26]–[29]. Taken together, a comprehensive set of behavioral assays allows for the analysis of larval behavior from naïve responses to higher brain functions.

Genetic manipulations have been widely used to elucidate the functions of neural circuits in larval behavior. The GAL4/UAS system allows for a convenient and reproducible expression of effector genes in defined subsets of cells [30]–[33]. The transcription factor GAL4, whose spatial and temporal expression is controlled by a flanking enhancer, determines the expression of the effector. For example, effectors that block neurotransmitter release or induce cell death have been used to impair neural function [34], [35]. In this study we have used a combination of the apoptosis inducing genes *head involution defective (hid*) [36], [37] and *reaper (rpr*) [38] to reliably ablate most of the neurons expressing serotonin (5-hydroxytryptamine, 5HT), using specific driver lines named TPH-GAL4 [39] and TRH-GAL4 [40]. Both of them utilize promoter fragments of the same *tryptophan hydroxylase* (Trh) gene to direct GAL4 expression to the 5HT system, as TRH was reported to catalyse the rate-limiting step of 5HT synthesis from tryptophan to 5-hydroxy-tryptophan [41]. It has to be mentioned that the nomenclature is rather confusing as the *Drosophila* genome harbors two different genes that both provide enzymatic activity to hydroxylate tryptophan. However, the initially described gene CG7399 (also called TPH, PAH, DTPH, Trh, Henna and DTPHu) is expressed in larval dopaminergic neurons and not in serotonergic neurons of the brain [42]. Only the later identified gene CG9122 (also called TRH, DTRHn) is expressed in the serotonergic neurons of the brain [42]. Unfortunately, although clearly distinct in their expression and even function, both genes are sometimes called TPH, similar to their conserved mammalian counterparts TPH1 and TPH2. Subject of this study is the gene CG9122 that can be functionally addressed by TPH-GAL4 and TRH-GAL4.

5HT is a biogenic amine, which are important neuroactive molecules in the central nervous system (CNS) of insects [41], [43], [44]. Apart from 5HT, the biogenic amines dopamine (DA), histamine (HA), tyramine (TA) and octopamine (OA) have been studied in *Drosophila.* Each of them consists of a stereotypic pattern of a small number of neurons that are widely distributed in the adult and larval CNS [41]. However, studies that provide a detailed description of these systems on the single-cell level are rather limited [19], [45]–[49].

Initial work was based on antibodies that specifically bind 5HT and thereby describe the larval 5HT system in general [50]–[52]. These studies showed that serotonergic neurons are mostly interneurons found in bilateral clusters in the CNS, in the feeding apparatus as well as in the major endocrine organ of the larva, the ring gland. Neither the number nor the projection patterns of these neurons seem to change significantly during larval development [50]. Within the CNS, the 5HT system consists of about 84 neurons, distributed in clusters of one to five neurons each [50]. Four distinct clusters can be recognized per brain hemisphere, called SP1, SP2, LP1 and IP containing about three, four, four and two neurons, respectively. The suboesophageal ganglion (SOG) includes three additional 5HT-positive clusters called SE1, SE2 and SE3; they comprise about two, three and three serotonergic neurons, respectively, per side. The three thoracic neuromeres T1, T2 and T3 contain about two 5HT neurons per side, except for T1 that consists of three neurons. The abdominal neuromeres A1-A7 include about two serotonergic neurons per side, whereas the fused terminal A8/A9 neuromere contains a single 5HT neuron per side [41], [50], [52]. It has to be mentioned that the nomenclature of the different clusters is partially misleading as the cell bodies of the thoracic and abdominal neurons are not located in the respective neuromere, only their projections innervate the respective brain area. However, although we are aware of this problem we will use the established nomenclature for this study.

Regarding the detailed anatomy of these neurons, two papers provided first insights [53], [54]. Roy and coworkers described a pair of contralaterally projecting serotonin-immunoreactive deutocerebral (CSD) interneurons (one per hemisphere) of the IP cluster [53]. In the larva, these neurons innervate both antennal lobes (AL) and the lateral protocerebrum and after metamorphosis expand their expression to the mushroom body (MB) calyx and lateral horn. Similar 5HT-positive large-field neurons have been described in a variety of insects. Functionally it was suggested that mechanosensory stimulation (for example, air currents) could trigger serotonin release from the CSD neurons to set the threshold of detection of odorants [55]–[58]. The second study used the flp-out technique to label single 5HT neurons in the abdominal ganglion, focusing on the A1–A7 two-cell 5HT clusters [54]. However, while these authors were able to describe the detailed morphology of these neurons, a comprehensive description of the 5HT system on the single-cell level is still lacking.

On the functional level, early studies that were based mainly on the innervation pattern of 5HT-positive neurons in pharyngeal muscles, proventriculus, midgut and ring gland suggested their modulatory role in larval feeding behavior and neuroendocrine activity [50], [52]. Recent studies addressed the effect of 5HT more directly by genetic interference and suggested a role of this system in larval light-dependent locomotion [59] as well as in olfaction, feeding and heart rate regulation [42], [60]. In addition, 5HT may also have developmental effects as it was suggested to regulate the density of varicosities [61] and the branching of 5HT-positive neurons [62].

In this study we first comprehensively describe the basic 5HT-positive neuronal network up to the single-cell level using the flp-out technique [63]. In the second part we analyze the necessity of the larval 5HT-positive neurons for innate olfactory-, gustatory- and visually-guided behaviors as well as appetitive and aversive olfactory learning. Taken together, we demonstrate that the serotonergic neuronal network mainly consists of interneurons that are not required *per se* for the basic behaviors addressed above but – based on other studies – may rather modulate some of these behaviors similar to the situation in mammals.

## Results

### General Anatomy of the Serotonergic System in the Larval CNS

**Figure 1:**
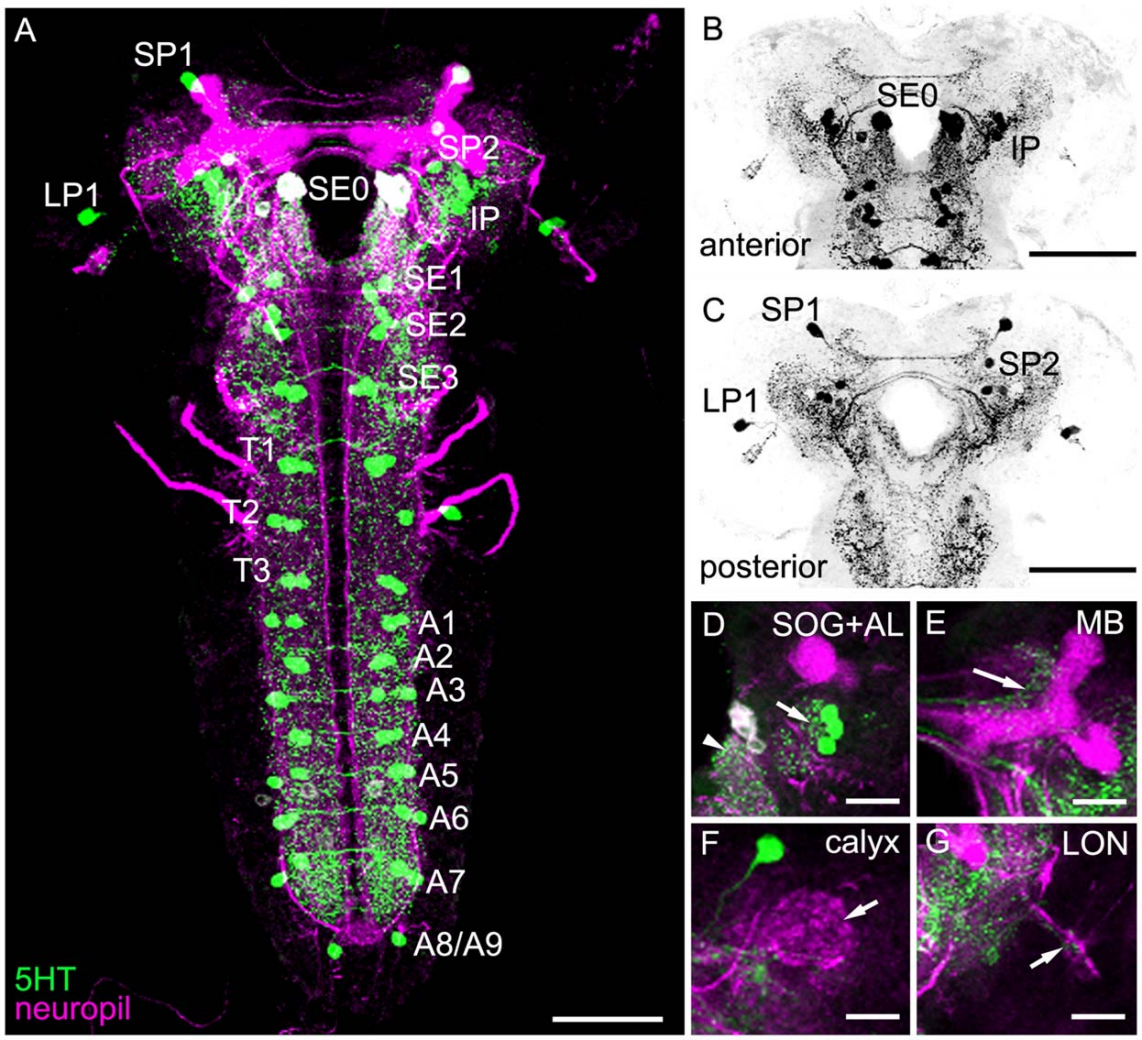
Anatomy of the Serotonergic System in the Larval CNS Based on anti-5HT Staining. 5HT positive cells (green) of Canton-S wild type larvae are shown in combination with anti-FasciclinII (FasII)/anti-Cholineacetyltransferase (ChAT) neuropil markers (magenta) (A and D–G). (A) The CNS of the third instar larva comprises 19 different 5HT-positive bisymmetrical clusters of one to three cells each. (B–G) In the brain hemispheres, five serotonergic clusters, SP1, SP2, LP1, SE0 and IP, were detected (in B and C only the anti-5HT channel is shown). (D) 5HT cells innervate the antennal lobe (AL; right arrow) and the suboesophageal ganglion (SOG; left arrowhead). (E) The mushroom body lobes (MB; arrow) and the (E) MB calyx (arrow) show only very week – if any - innervation. (F) By contrast, the larval optic neuropil (LON; arrow) is innervated by serotonergic arborizations. (B) and (C) show a frontal view of the anterior or posterior half of the brain, respectively. In (D–G) lateral is always to the right and medial to the left. Scale bars: A–C: 50 µm; D–G: 25 µm.

**Figure 2:**
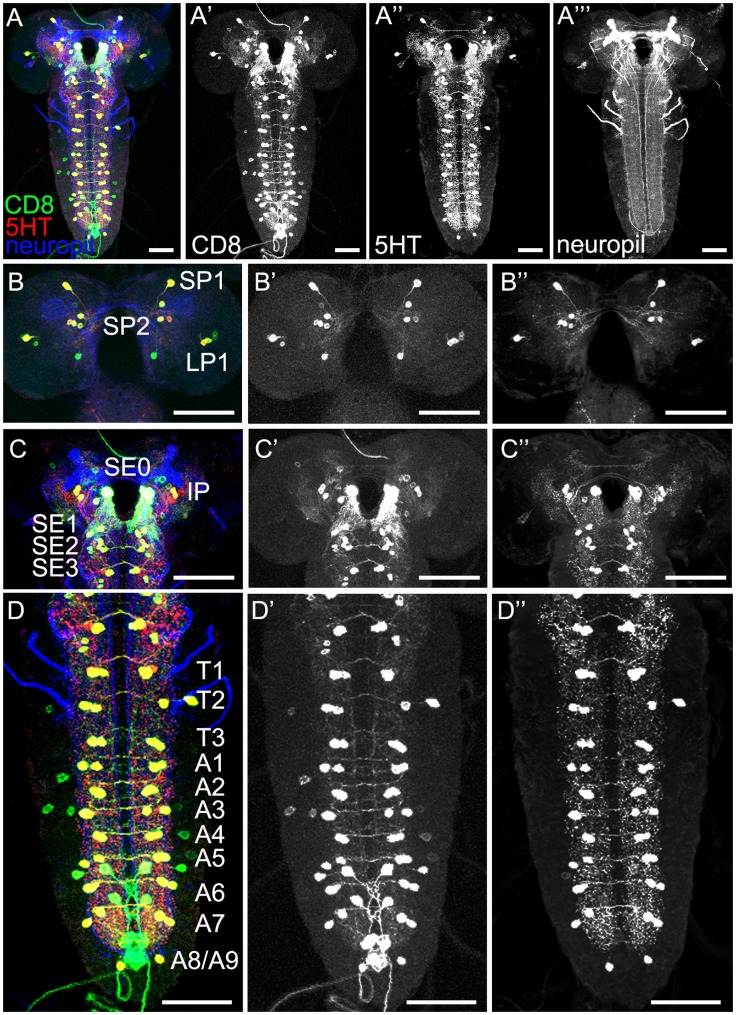
Expression Pattern of the Driver Line TRH-GAL4 in the Larval CNS. Triple staining of TRH-GAL4/UAS-mCD8::GFP third instar larvae in the first column shows cell membrane-bound CD8 labeling (green) combined with 5HT-immunoactivity (red) and anti-FasII/anti-ChAT staining for visualizing the neuropil (blue). The second (CD8), third (5HT) and fourth columns illustrate the three channels separately. The first row (A–A’’’) shows the whole CNS. The other rows represent higher magnifications of the brain in frontal view (B–B’’: posterior; C–C’’: anterior) and the ventral nerve cord (VNC) (D–D’’). A high co-localization of CD8- and 5HT-positive cells is found in the posterior hemisphere clusters SP1, SP2 and LP1 (B–B’’) as well as in the anterior clusters IP and SE0-3 (C–C’’). Nearly all cells of the VNC clusters T1-3 and A1–A8/A9 (D–D’’) show anti-CD8 and anti-5HT double staining. In addition some non-serotonergic CD8-expressing cells were detected (asterisks). Scale bars: 50 µm.

**Figure 3:**
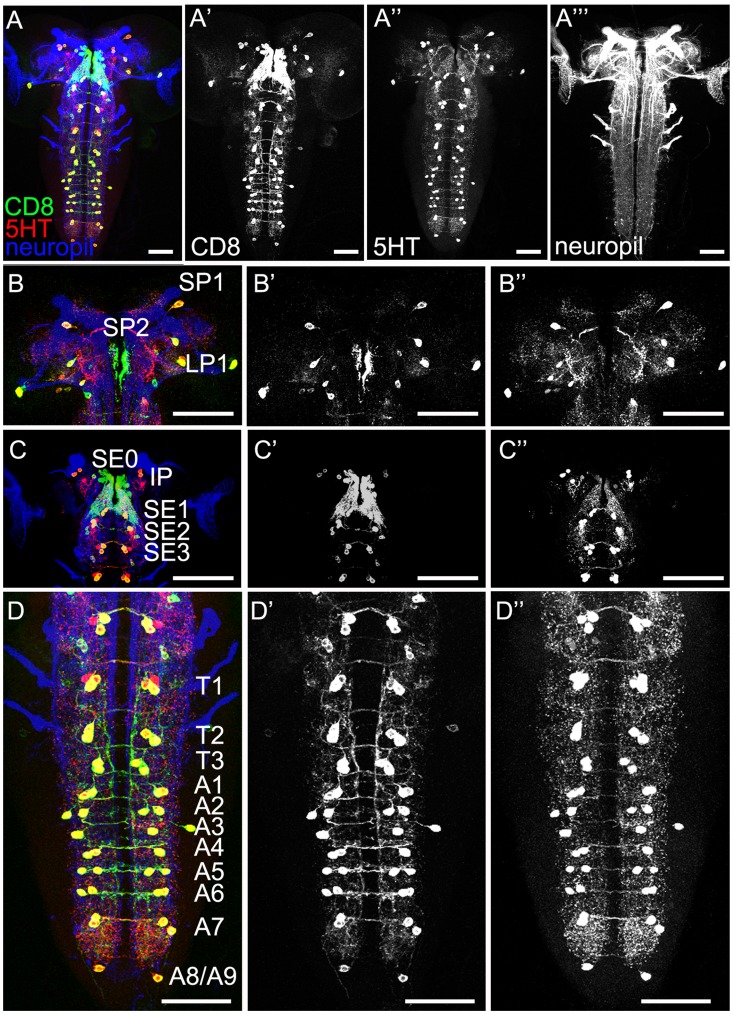
Expression Pattern of the Driver Line TPH-GAL4 in the Larval CNS. First column: CNS of TPH-GAL4/UAS-mCD8::GFP third instar larvae stained with anti-CD8 (green), anti-5HT (red) and anti-FasII/anti-ChAT (neuropil markers; blue). The second, third and fourth columns represent the three channels separately. The first row (A–A’’’) shows an overview of the CNS. Other rows represent higher magnifications of the brain in frontal view with slightly shifted brain hemispheres (B–B’’: posterior; C–C’’: anterior) and the ventral nerve cord (VNC) (D–D’’). In the posterior (B–B’’) and anterior brain (C–C’’) as well as in the VNC (D–D’’) most cells are both anti-CD8 and anti-5HT positive. Only in some clusters (e.g. SE3 and T1) a few CD8-positive cells do not show 5HT expression. Details are also presented in Table 1. Scale bars: 50 µm.

**Figure 4:**
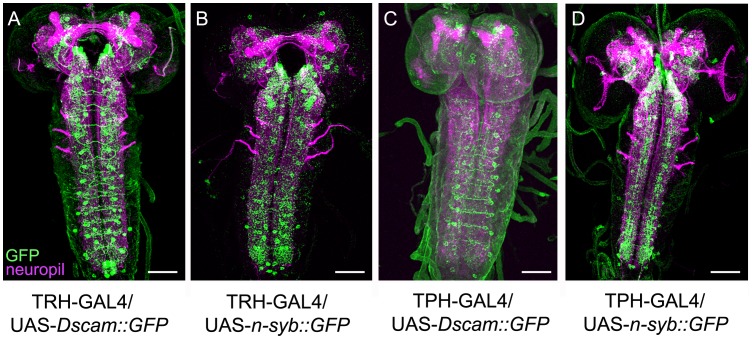
Post- and Presynaptic Organisation of the Larval Serotonergic System. By crossing TRH-GAL4 (A,B) and TPH-GAL4 (C,D) with UAS-*Dscam17.1::GFP* and UAS-*n-syb::GFP*, postsynaptic and presynaptic regions, respectively, were visualized. The brains of third instar larvae were stained with anti-GFP (green) and with anti-FasII/anti-ChAT (magenta). The expression patterns for the postsynaptic innervation are similar for the two driver lines, the same is true for the presynaptic labeling. Scale bars: 50 µm.

For analyzing the gross anatomy of the larval 5HT system with respect to the published data, we first used a 5HT-specific antibody in combination with anti-FasciclinII (FasII)/anti-Cholineacetyltransferase (ChAT) to visualize the 5HT-positive cells and neuropil structures at the same time (Figure 1) [5], [17], [19]. Second, we used the two GAL4 driver lines TRH-GAL4 and TPH-GAL4 for expressing UAS-mCD8::GFP (Figures 2 and 3) [39], [64]–[66] in order to use a triple staining protocol in which anti-FasII/anti-ChAT, anti-serotonin (5HT) and anti-CD8 (CD8) label at the same time the neuropil structures, 5HT cells and UAS-mCD8::GFP-positive cells, respectively. Triple staining allowed us to directly trace 5HT cells within the GAL4 pattern of the respective GAL4 line and to follow their projections within the neuropil (Table 1). Third, we further analyzed the 5HT system by expressing post- and presynaptic markers via TRH-GAL4 and TPH-GAL4, reflecting potential input and output sites of these neurons, respectively. Similar to our previous study of the DA system, we used the two effectors Dendrite-Specific Drosophila Down Syndrome Cell Adhesion Molecule, conjugated to GFP (Dscam[17.1]::GFP) (postsynaptic; Figure 4A and 4C) and neuronal synaptobrevin, conjugated to GFP (n-syb::GFP) (presynaptic; Figure 4B and 4D) [67], [68]. Overall, the larval 5HT system seems to establish dendritic innervations in the protocerebrum, SOG, AL as well as the thoracic and abdominal ganglia. Within the protocerebrum, expression tended to be denser within the medial parts compared to the lateral ones. 5HT arborizations in the larval optic neuropil (LON) do not seem to be dendritic, and there was no signal detectable within the MBs (Figures 1,2, 3, 4). Regarding the presynaptic organization, we detected massive expression throughout the protocerebrum lacking any particular pattern. In both lines, presynaptic staining was also visible in the LON, and especially in the SOG, thoracic and abdominal ganglia (Figure 4). Again MB lobes and calyces were not included in the expression pattern. Interestingly, the ALs were not labeled by any line crossed with UAS-n-syb::GFP (Figure 4). Therefore either expression was not detectable due to missing or week staining within the AL-specific CSD neurons, or more probable the CSD neurons are exclusively postsynaptic in the ALs. The opposite seems to be true for the 5HT-positive neurons innervating the LON (Figure 4). Thus, the larval visual system may only get input from the 5HT system but lacks direct output onto it.

**Table 1:** Cell numbers of potential serotonergic neurons in the larval nervous system.

Neuropil(Literature)	Literature*	anti-5HT(this study)	TrH-GAL4			TPH-GAL4			Neuropil(this study)
			CD8 positive	5HT positive	overlay	CD8 positive	5HT positive	overlay	
SP1	1**	1.3±0.7 (10)	1.1±0.3 (10)	1.3±0.5 (10)	1.0±0.0 (10)	1.0±0.0 (10)	1.3±0.7 (10)	1.0±0.0 (10)	SP1
SP2	4	3.6±0.5 (10)	4.3±0.5 (10)	3.4±0.4 (10)	3.3±0.4 (10)	5.0±0.5 (10)	3.6±0.5 (10)	3.8±0.9 (10)	SP2
IP	3–4	3.5±0.7 (10)	6.6±0.8 (10)	3.9±1.1 (10)	2.9±1.1 (10)	4.5±0.7 (10)	3.5±0.7 (10)	2.3±0.4 (10)	IP
LP1	2	2.1±0.3 (10)	3.5±0.5 (10)	2.1±0.3 (10)	1.0±0.0 (10)	1.3±0.3 (10)	2.1±0.3 (10)	1.0±0.0 (10)	LP1
n.d.	n.d.	3.5±0.5 (10)	3.7±0.7 (10)	2.9±0.6 (10)	2.5±1.1 (10)	6.1±1.3 (10)	2. 1±0.5 (10)	2.1±0.5 (10)	SE0
SE1	2	2.0±0.0 (10)	2.0±0.0 (10)	2.0±0.0 (10)	2.0±0.0 (10)	2.0±0.0 (10)	2.0±0.0 (10)	2.0±0.0 (10)	SE1
SE2	3	4.2±1.2 (10)	4.5±0.5 (10)	4.8±1.4 (10)	4.5±0.5 (10)	4.1±0.4 (10)	5.4±0.5 (10)	3.8±0.6 (10)	SE2
SE3	3	4.3±1.4 (10)	3.7±0.5 (10)	5.0±1.1 (10)	3.7±0.5 (10)	5.3±2.1 (10)	4.8±0.7 (10)	3.1±0.9 (10)	SE3
T1	3	3.0±0.0 (10)	2.2±0.4 (10)	3.0±0.0 (10)	2.0±0.0 (10)	2.0±0.0 (10)	3.0±0.0 (10)	2.0±0.0 (10)	T1
T2	2	2.0±0.0 (10)	5.3±0.5 (10)	2.0±0.0 (10)	2.0±0.0 (10)	2.0±0.0 (10)	2.0±0.0 (10)	2.0±0.0 (10)	T2
T3	2	2.0±0.0 (10)	5.1±0.6 (10)	2.0±0.0 (10)	2.0±0.0 (10)	2.0±0.0 (10)	2.0±0.0 (10)	2.0±0.0 (10)	T3
A1	2	2.0±0.0 (10)	2.0±0.0 (10)	2.0±0.0 (10)	2.0±0.0 (10)	2.0±0.0 (10)	2.0±0.0 (10)	2.0±0.0 (10)	A1
A2	2	2.0±0.0 (10)	2.0±0.0 (10)	2.0±0.0 (10)	2.0±0.0 (10)	2.0±0.0 (10)	2.0±0.0 (10)	2.0±0.0 (10)	A2
A3	2	2.0±0.0 (10)	4.5±0.8 (10)	2.0±0.0 (10)	2.0±0.0 (10)	2.0±0.0 (10)	2.0±0.0 (10)	2.0±0.0 (10 )	A3
A4	2	2.0±0.0 (10)	3.4±0.7 (10)	2.2±0.3 (10)	2.0±0.0 (10)	2.0±0.0 (10)	2.0±0.0 (10)	2.0±0.0 (10)	A4
A5	2	2.2±0.3 (10)	3.3±0.6 (10)	2.2±0.3 (10)	2.0±0.0 (10)	2.0±0.0 (10)	2.2±0.3 (10)	2.0±0.0 (10)	A5
A6	2	2.1±0.2 (10)	4.3±0.7 (10)	2.4±0.4 (10)	2.0±0.0 (10)	2.0±0.0 (10)	2.1±0.2 (10)	2.0±0.0 (10)	A6
A7	2	2.0±0.0 (10)	3.5±0.7 (10)	2.0±0.0 (10)	2.0±0.0 (10)	2.0±0.0 (10)	2.0±0.0 (10)	2.0±0.0 (10)	A7
A8	1	1.0±0.0 (10)	4.5±0.5 (10)	1.0±0.0 (10)	1.0±0.0 (10)	1.8±0.6 (10)	1.0±0.0 (10)	1.0±0.0 (10)	A8
Brain	10–11	10.5±1.1 (10)	15.5±2.2 (10)	10.7±2.3 (10)	8.2±1.5 (10)	11.8±1.5 (10)	10.5±2.2 (10)	8.1±1.3 (10)	Brain
SOG	8	16.0±3.1 (10)	13.9±1.6 (10)	14.7±2.1 (10)	12.7±2.1 (10)	17.5±3.8 (10)	14.3±1.7 (10)	11.0±2.0 (10)	SOG
Thoracic ganglion	7	7.0±0.0 (10)	12.6±1.5 (10)	7.0±0.0 (10)	6.0±0.0 (10)	6.0±0.0 (10)	7.0±0.0 (10)	6.0±0.0 (10)	Thoracic ganglion
Abdominal ganglion	15	15.4±0.4 (10)	27.5±4.1 (10)	15.8±1.0 (10)	15.0±0.0 (10)	15.8±0.6 (10)	15.3±0.5 (10)	15.0±0.0 (10)	Abdominal ganglion
TOTAL	40–41	48.9±3.5 (10)	69.5±9.4 (10)	48.2±5.4 (10)	41.9±3.6 (10)	51.1±5.9 (10)	47.1±4.4 (10)	40.1±3.3 (10)	TOTAL

all numbers refer to clusters or brain regions in one brain hemisphere.

* Valles and White (1988).

** Valles and White described three neurons in SP1. However, two of them only show up after larval life.

Thus, we changed the number to one, thereby only describing the developmental stage of the larvae.

**Figure 5:**
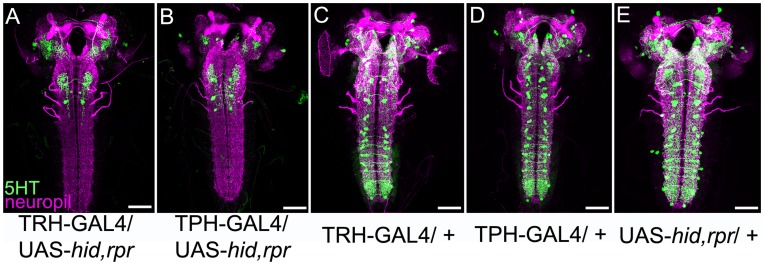
Ablation of the Serotonergic Neurons via UAS-*hid,rpr* Expression. UAS-*hid,rpr* (*head involution defective*; *reaper*) was crossed with TRH-GAL4 (A) or TPH-GAL4 (B) and stained with anti-5HT (green) and anti-FasII/anti-ChAT (magenta). Nearly all serotonergic neurons undergo apoptosis. Only a small number of 5HT cells in the VNC, the hemispheres and the SOG were not ablated by *hid* and *reaper* expression. A similar expression pattern compared to wild type (Figure 1) was detectable in all control groups, by crossing either the two GAL4-lines (C, D) or the UAS-line (E) with *white^1118^* control flies. Scale bars: 50 µm.

In addition we crossed TRH-GAL4 and TPH-GAL4 with the apoptosis-inducing effectors UAS-*hid,rpr* to induce directed cell death within the 5HT system. Again, we applied anti-FasII/anti-ChAT antibodies to label the neuropil and anti-5HT to visualize the remaining serotonergic neurons (Figure 5). The results essentially support the finding that the two GAL4 lines cover nearly all 5HT neurons, although a small fraction of 5HT neurons was still visible in the experimental larvae (Figure 5A and 5B). Thus, both lines allow for ablation of nearly all 5HT neurons. However, the persistence of several neurons of the same type in both approaches affects the interpretation of the behavioral experiments to some respect (see also Discussion).

These data were useful to get a general idea of the organization of the 5HT system. However, they did not allow us to reconstruct the morphology of individual serotonergic neurons. To this end, we applied a single-cell approach based on the flp-out technique [63]. In combination with the triple staining protocol mentioned above we were thus able to randomly induce single-cell clones of the different 5HT positive cells. Based on more than 500 preparations, we describe here the morphology of 5HT cells that were independently hit in at least two different preparations, most often in both GAL4 lines. In the following, we present the different cell clusters, from the brain region to the last abdominal neuromere (summarized in Table 1). The brain neuropil nomenclature used to describe the projection patterns of the single serotonergic neurons is based on Selcho et al. (2009).

### Anatomy of the Serotonergic System: Brain Hemispheres

**Figure 6:**
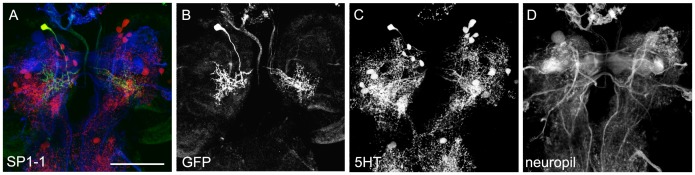
Morphology of the SP1 Cell. SP1-1 type 5HT cell as shown in single-cell flp-out clones via anti-CD8 (green), anti-5HT (red) and anti-FasII/anti-ChAT (blue) staining (A). The three channels of the staining are presented individually in panels B–D. Scale bar: 50 µm.

In the **SP1** cluster, only a single cell was labeled called SP1-1, both by the 5HT antibody and the two GAL4 lines. In both of them, there was a perfect colabeling of this cell by the antibody (Table 1; Figures 1G, 2B–B’’ and 3B–B’’). Interestingly, Vallés and White (1988) reported a SP1 cluster consisting of three cells. However, since two of these cells can only be detected by midpupal stage, both observations are in agreement. The detailed morphology of the larval SP1 neuron is shown in Figure 6. A neurite projected from the dorsoposterior cell body basal halfway through the brain. It bifurcated in the posterior basomedial part of the brain and broadly innervated the basomedial brain hemispheres both ipsi- and contralaterally.

**Figure 7:**
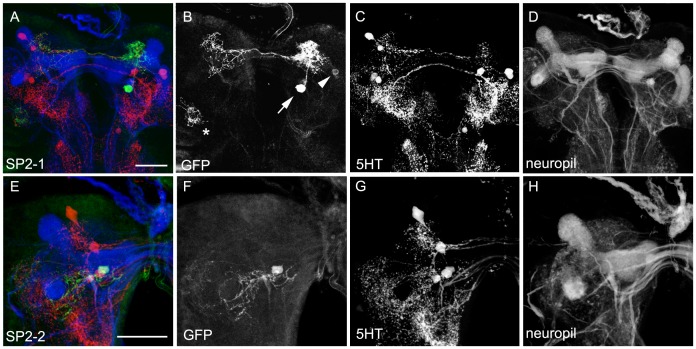
Morphology of the SP2 Cells. SP2-1 and SP2-2 type 5HT cells shown in single-cell flp-out clones via anti-CD8 (green), anti-5HT (red) and anti-FasII/anti-ChAT (blue) staining (A and E). The three channels are presented individually in panels B–D and G–H. In A and B three cells are labeled by the flp-out technique. Besides the SP2-1 cell (arrow), weak expression was detectable in an additional cell body (arrowhead) and a third cell of the LP cluster (asterisk). The SP2-2 cell was only weakly labeled and therefore likely misses a comprehensive visualization of its entire morphology. Scale bars: 25 µm.

The **SP2** cluster consisted of three to four 5HT-immunoreacitive cells and was located posterior to the medial lobes of the MB. For all neurons we noted a strongly variable cell body position in between specimens. Although TRH-GAL4 and TPH-GAL4 expressed CD8 in about five cells of the cluster only three cells showed co-labeling with anti-5HT in both lines. Thus both lines overlapped only partially with the 5HT cells of the SP2 cluster and labeled additional cells (Table1; Figure 1C, 2B–B’’ and 3B–B’’). Two serotonergic GAL4-positive cells were reliably stained in the cluster (Figure 7). The first one, called SP2-1, projected ipsilaterally and innervated the dorsomedial and basomedial brain next to the vertical MB lobe. From there, neurites extended contralaterally to innervate sparsely the dorsomedial and basomedial part of the brain (Figure 7A–D). The second SP2 cell, called SP2-2, innervated mainly the basolateral protocerebrum. One of its processes projects around the MB peduncle and another one faintly innervated the lateral part of the brain. Due to low CD8 expression levels we are not sure if the cell was completely represented, although two further clones suggested a similar innervation pattern. The third GAL4 positive cell of both driver lines was not hit by flp-out. It therefore remains unknown, like the additional 5HT cell that is not included in the expression pattern of both GAL4 lines.

**Figure 8:**
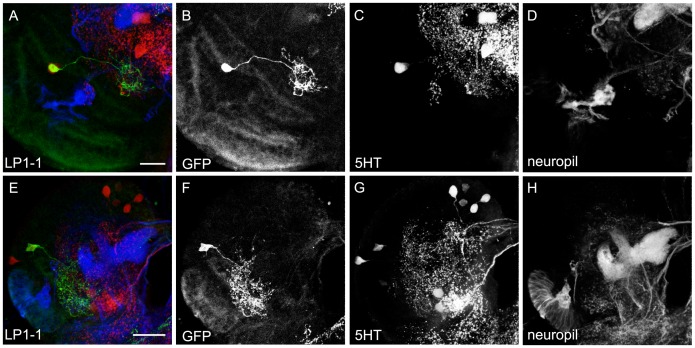
Morphology of the LP1 Cells. LP1-1 type 5HT cells shown in single cell flp-out clones via anti-CD8 (green), anti-5HT (red) and anti-FasII/anti-ChAT (blue) staining (A and E). The three channels are presented individually in panels B–D and G–H. Two examples for different flp-out clones are shown in A and E. Due to the variation in their morphology it is not possible to clarify, if the two clones label the same cell or two different cells of the LP cluster. Thus, in more restricted manner we categorized both clones as LP1-1. Scale bars 10 µm (in A) and 25 µm (in E).

The third posterior cluster of 5HT cells within the larval brain is called **LP1**. Two of its cells were reported to have their soma in the medial lateral cortex [50]. Again both of our GAL4 lines lacked one 5HT cell in the cluster and overlapped with only one cell. Furthermore, TRH-GAL4 showed extra expression in about three non-5HT cells (Table1; Figures 1G, 2B–B’’’ and 3B–B’’’). Figure 8 depicts the morphology of the two 5HT cells found in both lines. However, as it is not clear if the patterns shown refer to a single neuron that shows variable morphology between different individuals or if they reflect two neurons which are both characterized by a basolateral cell body and projections medially to the basolateral brain area (Figure 8A–H), we call both cells LP1-1.

**Figure 9:**
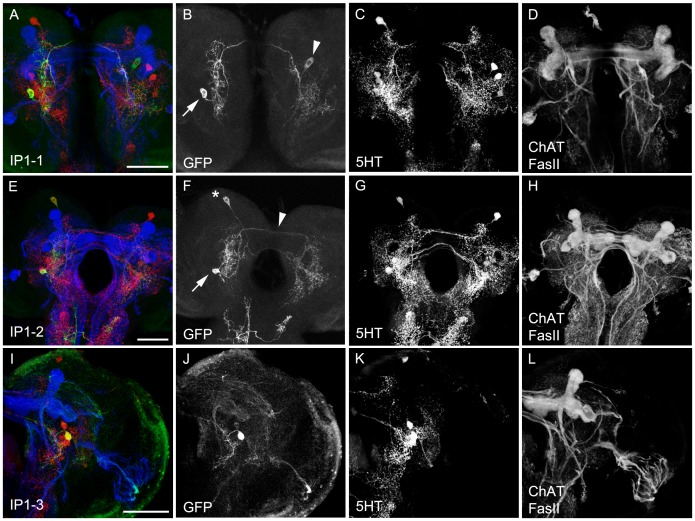
Morphology of the IP Cells. IP1-1, IP1-2 and IP1-3 type 5HT cells shown in single-cell or two-cell flp-out clones via anti-CD8 (green), anti-5HT (red) and anti-FasII/anti- ChAT (blue) staining (A, E and I). The three channels are presented individually in panels B–D, G–H and J–L. The IP1-1 cell (B, arrow) is visualized in a double flp-out clone that shows an additional weakly labeled cell body in the right hemisphere (arrowhead). The IP1-2 cell (F) is also visualized in a double flp-out clone together with the SP1-1 cell (see also Figure 6). The arrow marks the cell body of the IP1-2 cell that innervates the ipsi- and contralateral hemispheres by crossing the midline more dorsal (arrow) compared to the SP1-1 cell that crosses the midline next to the pharynx. The expression in the SOG belongs to third cell of a different type that does not innervate the brain hemispheres. Scale bars 25 µm.

The last cluster of 5HT cells in the brain described by Vallés and White (1988), called **IP**, is located anterior basomedial between the AL and the lateral appendix of the MB [18]. It consists of about four 5HT cells three of which overlapped with TRH-GAL4 and TPH-GAL4 (Table1; Figures 1B, 2C–C’’ and 3C–C’’). When expressing the apoptosis-inducing genes *hid* and *rpr* in both GAL4 lines [36]–[38], the persisting cells suggested that for TRH-GAL4 (n = 10) the CSD neuron that we call IP1-1 [53] is not targeted (Figure 5). However, flp-outs of TRH-GAL4 hit the CSD neuron three times (Figure 9A–9D), thus indicating variation in the cell ablation experiments. From the cell body located anterior of the dorsal basolateral protocerebrum, a primary neurite projected to the posterior end of the AL and either innervated the AL itself and sometimes its adjacent area. From there a single process extended dorsoposteriorly along the antennocerebral tract (ACT) and split well before reaching the MB calyx. Its extensive arborizations innervated a region between the lateral and medial part of the posterior brain. A single neurite passed further from the ipsilateral ACT via the midline to the contralateral ACT, establishing a few terminal branches. However, its major processes followed the ACT and innervated the contralateral AL. A second type of IP neuron, called IP1-2 (shown in Figure 9E–H in a double clone together with a SP1 cell) showed a similar ipsilateral but a different contralateral morphology. It massively branched in posterior dorsolateral and anterior basolateral brain areas and thus could be clearly distinguished from the IP1-1 neuron (Figure 9E-H). The third IP neuron, called IP1-3 was hit five times but expression levels were always very low, excluding its detailed description. Nevertheless, from the anterior basomedial located cell body a primary neurite projected posteriorly, bifurcated and innervated basomedial brain regions (Figure 9I-L). Due to the low expression levels, it is not clear if the second branch crosses the midline and innervates contralateral brain areas similar to the other two IP cells.

Taken together, from the approximately ten to eleven 5HT positive neurons reported initially in one brain hemisphere (two cells of the SP2 cluster appear later in metamorphosis) [50], we were able to identify eight reliably within the expression patterns of both GAL4 lines. The detailed evaluation of TRH-GAL4 and TPH-GAL4 revealed that the expression patterns of the two lines include besides the about eight serotonergic cells additional expression in a small set of 5HT-negative neurons (Table 1). On the single-cell level we were able to identify about seven of these cells. This suggests that we only miss a single cell type of the SP2 cluster and the 5HT positive cells that might not be included in the Gal4 lines in our analysis. Unfortunately, this neuron, which likely projects into the LON, was not revealed by any single-cell clone.

### Anatomy of the Serotonergic System: Suboesophageal Ganglion

Vallés and White (1988) reported that the larval SOG is organized by three bilaterally symmetrical clusters called SE1, SE2 and SE3. Apart from these clusters, we found an additional cluster of 5HT positive somata located anterior at the very tip of the SOG (Figures 1A, 1B, 2C–C’’, 3C–3C’’). Although the intensity of anti-5HT staining varied considerably within this cluster, it was obvious in most of our samples which justifies the introduction of new 5HT cluster. We termed it SE0, based on its position anterior to SE1.

The **SE0** cluster consisted of about three 5HT-positive cells all of which were included in the expression pattern of TRH-GAL4 (Table 1 and Figure 2C–C’’). On average, two of the three cells were included in the TPH-GAL4 pattern (Table 1 and Figure 3C–C’’). As TRH-GAL4; UAS-*hid,rpr* experimental larvae completely lacked the SE0 cluster (Figure 5) while a single 5HT-positive cell per hemineuromere persisted in TPH-GAL4; UAS-*hid,rpr* larvae (Figure 5), the cell ablation data confirmed the observations obtained by the triple staining approach (Figures 2 and 3). In the flp-out approach we only detected a single type of 5HT cell, called SE0-1 potentially due to a similar morphology of these neurons. It densely innervated the anterior end of the SOG and sent a fiber into the periphery that we were not able to follow. However, in all our samples it was not clear, if the cell is really serotonergic. Thus, we will not present the neuron in the manuscript to avoid any misunderstanding.

The **SE1** cluster comprised two 5HT-positive cells which were specifically labeled by both GAL4 lines (Table 1; Figures 1, 2C-C’’ and 3C-C’’). Remarkably, in this cluster there was no variation of the expression pattern of the GAL4 lines throughout the samples (Table 1). Unfortunately, in more than 500 clones we never hit these cells, precluding any detailed description.

For the **SE2** cluster, three 5HT cells have been reported by Vallés and White (1988). In contrast, we observed a total of four to five cells in this cluster. Three of them had their soma located anterior ventromedial. Two extra cell bodies, smaller in diameter, were located more posterolateral than the other somata (Figures 2C-C’’ and 3C-C’’). The obtained staining was in general quite weak and variable. TRH-GAL4 showed expression in nearly all of these cells (Table1; Figure 2C-C’’), whereas the TPH-GAL4 lines lacked one cell of the anterior medial cluster (Table1; Figure 3C-C’’).

**Figure 10:**
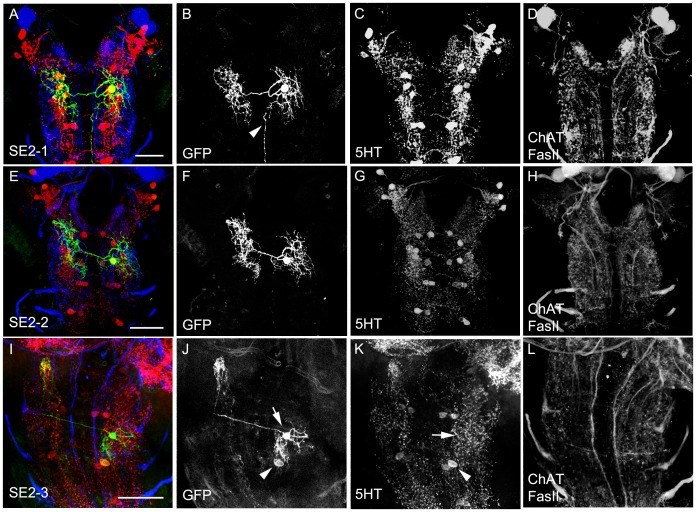
Morphology of the SE Cells. SE type 5HT cells shown in single cell flp-out clones stained via anti-CD8 (green), anti-5HT (red) and anti-FasII/anti- ChAT (blue) (A, E, I). The three channels are presented individually in panels B–D, F–H, J–L. The SE2-1 cell (A) is visualized by a single cell flp-out clone of the larval brain hemispheres; however there is an additional projection from an additional non-5HT descending neuron of the abdominal cluster (arrowhead). Similarly, the SE2-2 cell type bifurcated close to its cell body and sent branches ipsi- and contralaterally. The contralateral branch split again and its extensions covered the contralateral hemineuromere completely from ventral to dorsal. The SE2-3 cell (I-J) is shown as a double flp-out clone that also visualizes the cell body of an additional 5HT cell (arrowhead). The SE2-3 cell is only weakly labeled by anti-5HT (S, arrow). Scale bars 25 µm.

Like SE2, the **SE3** cluster comprised five 5HT-positive cells three of which had an anteriomedial and two a posteriolateral soma. Both GAL4 lines similarly labeled only four cells missing one cell that had its cell body located in the anteriomedial cluster. Again, these results were independently verified by our cell ablation approach, as in TRH-GAL4; UAS-*hid,rpr* and TPH-GAL4; UAS-*hid,rpr* larvae a single 5HT-positive neuron was left in the SE3 cluster (Figure 5). On the single-cell level, we obtained similar results for the SE2 and SE3 clusters and therefore only present the data for the former. Two obviously similar 5HT cells, called SE2-1 (Figure 10A-D) and SE2-2 (Figure 10E–H), bifurcated close to their cell body and sent branches ipsi- and contralaterally. The contralateral branch split again and its extensions covered the contralateral hemineuromere completely from ventral to dorsal (Figure 10A–H; the posterior projection in B shows and ascending fiber of a 5HT negative cell located in the abdominal ganglion). The ipsilateral branch divided less extensively and remained restricted to the ventromedial part of the hemineuromere. The two extra cells with smaller, posterolateral cell bodies were called SE2-3 and SE2-4 (Figures 2C–C’’ and 3C–C’’). Both cells had a similar morphology based on several double flp-out clones (Figure 10I–L and data not shown). A side branch of the primary neurite innervated the ventrolateral part of the neuromere, while a second side branch arborized in its dorsomedial portion before crossing the midline. The contralateral pattern consisted of a dorsal process extending laterally to the middle of the neuromere before turning sharply anterior and innervating a defined area of the proceeding neuromere.

In summary, the 5HT system of the SOG consists of four clusters called SE0, SE1, SE2 and SE3 comprising in total about 16 cells per hemineuromere. Both GAL4 lines lack some cells within the SOG (about two for TRH-GAL4 and three for TPH-GAL4) (Table1). While SE0 and SE1 clusters are organized differently, SE2 and SE3 show a similar pattern regarding cell body position and diameter. On the single-cell level, we were able to partially describe the serotonergic neurons for the SOG (except for SE0 and SE1).

### Anatomy of the Serotonergic System: Thoracic Ganglion

Regarding the thoracic ganglion three cell clusters were described located ventromedial close to each hemineuromere, called T1, T2 and T3 (the nomenclature refers to the respective thoracic hemineuromere, but see also the introduction for occurring problems) [41], [50], [52].

The **T1** cluster was reported to consist of three 5HT cells [50], a number which was confirmed by all of our samples (Figure 1). However, both TRH-GAL4 and TPH-GAL4 labeled only two cells, lacking expression or only weakly expressing in the third one (Table1; Figures 2D–D’’’ and 3D–D’’’). This result was again confirmed by the ablation procedure. Both GAL4 lines crossed to UAS-*hid,rpr* showed a single surviving 5HT-positive cells in T1 (Figure 5).

**Figure 11:**
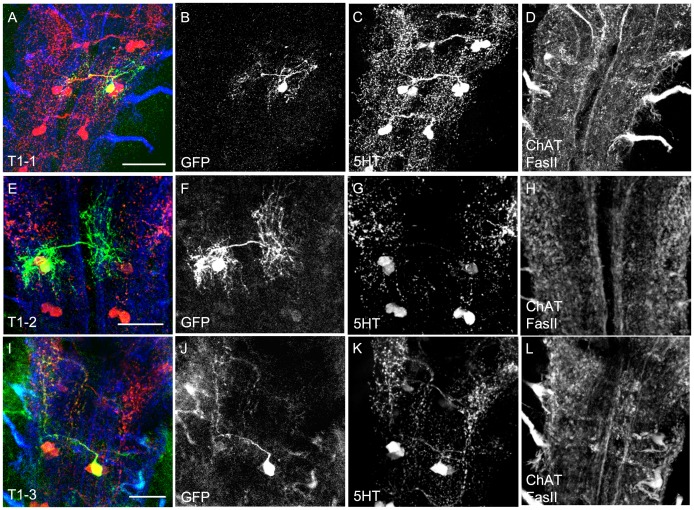
Morphology of the T1 Cells. 5HT cells in the T1 neuromere as shown in single cell flp-out clones via anti-CD8 (green), anti-5HT (red) and anti-FasII/anti- ChAT (blue) staining (A, E, and I). The three channels are presented individually in panels B–D, F–H and J–L. For the T1-3 cell (J) only limited information is presented due to the low quality of the GFP staining of the flp-out clone. Scale bars 25 µm.

The first of the T1 cells, called T1-1, branches next to the cell body (Figure 11A–D). The contralateral branch densely innervated the ventral T1 neuromere and its lateral margins. The ipsilateral branch split again; one branch extended to the dorsal neuromere border, the other to the midline which it followed both ipsi- and contralaterally. The second T1 cell – T1-2 (Figure 11E–H) - bifurcated into an ipsi- and a contralateral branch. The former split again and innervated the ipsilateral hemineuromere completely from ventral to dorsal. The latter established less extensive arborizations which were restricted to the ventromedial part of the hemineuromere. The third T1 neuron (T1-3) likely was anatomically similar to the small cells – SE2-3 and SE2-4 - of the SE2 and SE3 cluster (Figure 10I–L). However, the weak expression of GFP in all our samples limited its detailed anatomical description (Figure 11I–L).

**Figure 12:**
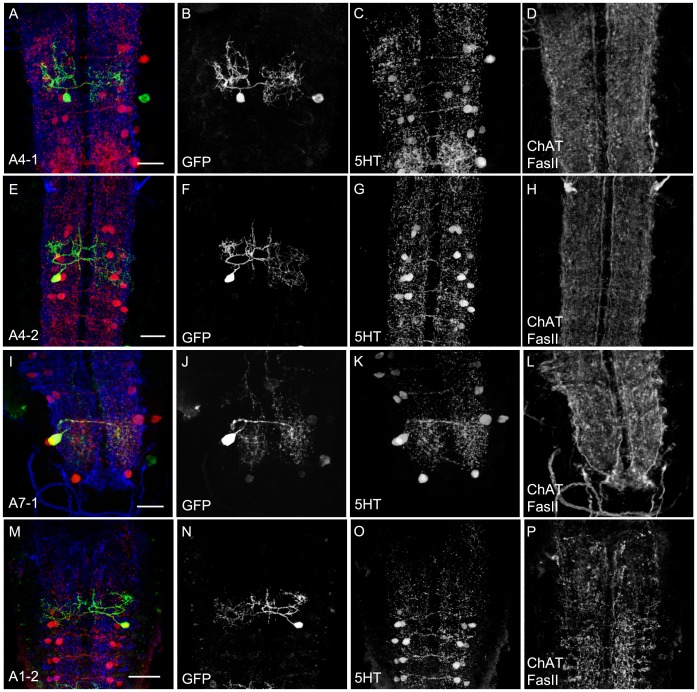
Morphology of the A1–A7 Cells. 5HT cells in A1–A7 neuromeres shown in single-cell flp-out clones via anti-CD8 (green), anti-5HT (red) and anti-FasII/anti- ChAT (blue) staining (A, E, I and M). The three channels are presented individually in panels B–D, F–H, J–L and N–P. Similar to Chen and Condron (2008) we were able to characterize to types of 5HT neurons for A1–A7 called type1 and type2. The two types of neurons are representatively depicted for A4-1 (A–D) and A4-2 (E–H). For the 5HT positive neurons innervating the outer neuromeres A1 and A7 there was a trend to restrict their innervation to the anterior (for A1) and posterior (A2) boarders. In B and J there are additional cell bodies labeled on a lower level. Scale bars 25 µm.

The clusters **T2** and **T3** comprised two 5HT cells each that were included in the expression pattern of both GAL4 lines (Table1; Figures 2D–D’’ and 3D–D’’). This pattern was confirmed by anti-5HT staining. Yet, while the expression pattern of TPH-GAL4 was restricted to these cells, TRH-GAL4 labeled three more cells that were 5HT-negative (Table1; asterisks in Figure 2D–D’’). In TPH-GAL4; UAS-*hid,rpr* ablated larvae, we still detected some of these cells by anti-5HT staining (Figure 5). This suggests either a counting error or different cell numbers for the triple staining approach and anti-5HT and anti-FasII/anti-ChAT staining. Alternatively, apoptosis induction may not be fully efficient due to low levels of *hid* and *rpr* expression. On the single-cell level, the morphology of these two cells is similar to the neurons described in detail below for the neuromeres A1–A7 (see Figure 12A–H).

Alltogether, the 5HT system in the larval thoracic neuromeres consists of about seven neurons that are mostly but not fully included in the expression pattern of the two GAL4 lines. While the T1 cluster consists of three cells, the hemineuromeres T2 and T3 comprise only two cells each of similar shape. On the single-cell level we were able to comprehensively describe all of these cells.

### Anatomy of the Serotonergic System: Abdominal Ganglion

**Figure 13:**
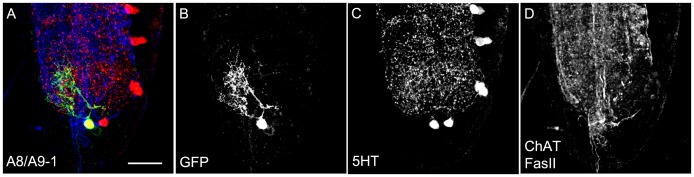
Morphology of the A8/A9 Cell. The single 5HT cell of the A8/A9 neuromere shown in a single cell flp-out clone via anti-CD8 (green), anti-5HT (red) and anti-FasII/anti- ChAT (blue) staining (A). The clone is not presented as a frontal view but rather as a sagittal view. The three channels are presented individually in panels B–D. Scale bar 25 µm.

According to Vallés and White (1988), the hemineuromeres A1 to A7 comprise two 5HT cells each, while the terminal fused A8/A9 neuromere includes only a single 5HT cell per side. These data were confirmed by Chen and Condron (2008) who described individual 5HT cells of the neuromeres A1 to A7 by using TPH-GAL4 in combination with the flp-out technique. Our own data from TPH-GAL4 and TRH-GAL4 demonstrating two cells in A1–A7 and a single one in A8/A9 (Figure 3D–D’’) are in agreement with these results. However, the TRH-GAL4 pattern included additional, 5HT-negative cells in neuromeres A3–A8/A9 (Table1; Figure 2D–D’’). Regarding the cellular anatomy we can refer to the detailed description of Chen and Condron for the medial and lateral neurons in A1–A7 (Figure 12A–H) that we called A1-1 and A1-2 (A2-1 and A2-2 and so on; in each case depending on its cell body position). In Figure 12 the morphology of the 5HT cells for the A4 is given (Figure 12A–H). In contrast to this organization, in the A7 neuromere the medial cell was characterized by a single primary branch that extended centrally and dispersed into a dense cloud of varicosities (Figure 12I–L). Furthermore, the 5HT cells in the A1 cluster tended to innervate its neuromere incompletely but instead partially invaded the T3 neuromere (Figure 12M–P). The primary neurite of the 5HT cell in the terminal A8/A9 neuromere bifurcated next to the cell body to send out a small ipsilateral primary branch (Figure 13). It densely innervated this area, together with the ipsilateral projections from A7 cells (Figure 13A–D).

In summary, both GAL4 lines cover all 15 5HT cells on each side of the eight abdominal neuromeres. This was also verified by the expression of *hid* and *rpr* via both lines that led to a full ablation of all 5HT-positive cells within the abdominal ganglion (Figure 5). Hence, we were able to individually describe the entire set of abdominal serotonergic neurons.

In conclusion, our comprehensive analysis of the two driver lines shows that these genetic tools are fairly specific for the larval 5HT system. This allowed us to genetically ablate the underlying neurons in order to address the role of 5HT for larval behavior. In particular, we analyzed naïve responses to odors, sugars, salt and light as well as learning and memory.

### TRH-GAL4 and TPH-GAL4 Positive Neurons are not Necessary for Overall Larval Olfactory Chemotaxis

For testing the role of 5HT-positive neurons in naïve olfactory preferences we placed 30 larvae onto a neutral only agarose filled Petri dish and let them chose for 5 minutes between an empty odor container and a container filled with AM. Other larvae were tested similarly for their innate preference for BA [19], [24].

**Figure 14:**
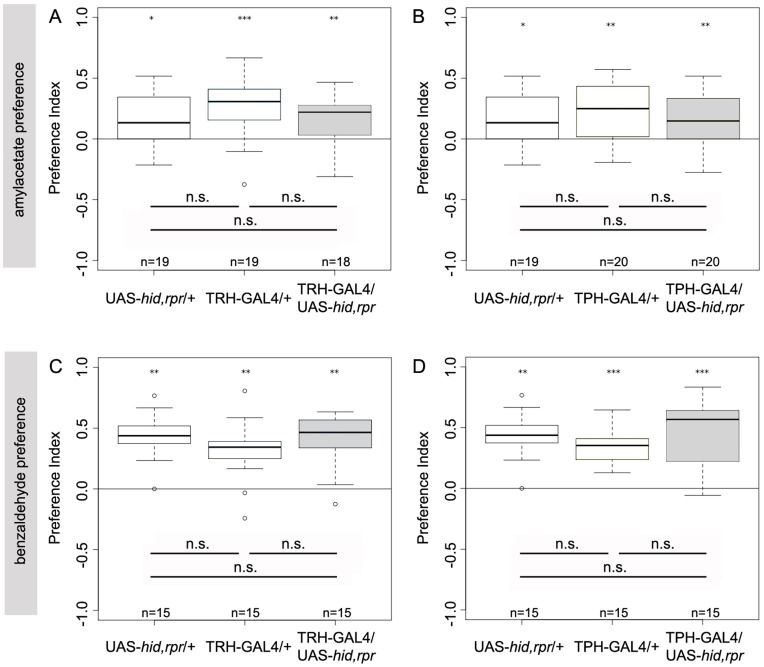
Serotonergic Neurons of the CNS are not Necessary for Olfactory Chemotaxis towards Amylacetate and Benzaldehyde. Third instar larvae with almost completely ablated serotonergic neurons were tested for naïve amylacetate (AM) (A, B) and benzaldehyde (BA) (C, D) preferences. TRH-GAL4/UAS-*hid,rpr* larvae showed preference for AM (p<0.01 compared to zero) (A) and for BA (p<0.01 compared to zero) (C). Compared to the controls UAS-*hid,rpr*/+ and TRH-GAL4/+, TRH-GAL4/UAS-*hid,rpr* did not perform significantly different either in AM or in BA preference tests (p>0.05). Similar results were found by testing TPH-GAL4/UAS-*hid,rpr* larvae. They preferred AM (p<0.01) (B) as well as BA (p<0.001) (D) and showed in both assays no significant difference to any control line (p>0.05). Under each boxplot of the figure for each genotype the sample size is shown; n = 15−20. Asterisks above each boxplot indicate, if the data is significantly different from zero. *<0.05; **<0.01; ***<0.001.

When AM was tested against no odor, TRH-GAL4/UAS-*hid,rpr* ablated larvae showed no reduction in their naïve AM preference when compared to either TRH-GAL4/+ or UAS-*hid,rpr*/+ control groups (Figure 14A; p = 0.09 and p = 0.75). Also TPH-GAL4/UAS-*hid,rpr* larvae in the same situation showed a preference that was not significantly different from both TPH-GAL4/+ and UAS-*hid,rpr*/+ controls (Figure 14B; p = 0.52 and p = 0.70). When testing for naïve BA preference against no odor, TRH-GAL4/UAS-*hid,rpr* ablated larvae did not perform significantly different form either TRH-GAL4/+ or UAS-*hid,rpr*/+ control larvae (Figure 14C; p = 0.18 and p = 0.98) and TPH-GAL/UAS-*hid,rpr* larvae also behaved indistinguishable from TPH-GAL4/+ or the UAS-*hid,rpr* control larvae (Figure 14D; p = 0.22 and p = 0.69). Therefore the larval serotonerigc system is not necessary for overall larval olfactory chemotaxis, at least in our test conditions.

### The Function of Serotonergic Neurons in Gustatory Chemotaxis

For testing if simple larval gustatory responses to sucrose, fructose and salt depend on 5HT signaling, we placed 30 larvae on a Petri dish that contained pure agarose on one half and the taste stimulus dissolved in agarose on the other half. Again larvae were allowed five minutes to chemotax [24], [69], [70].

**Figure 15:**
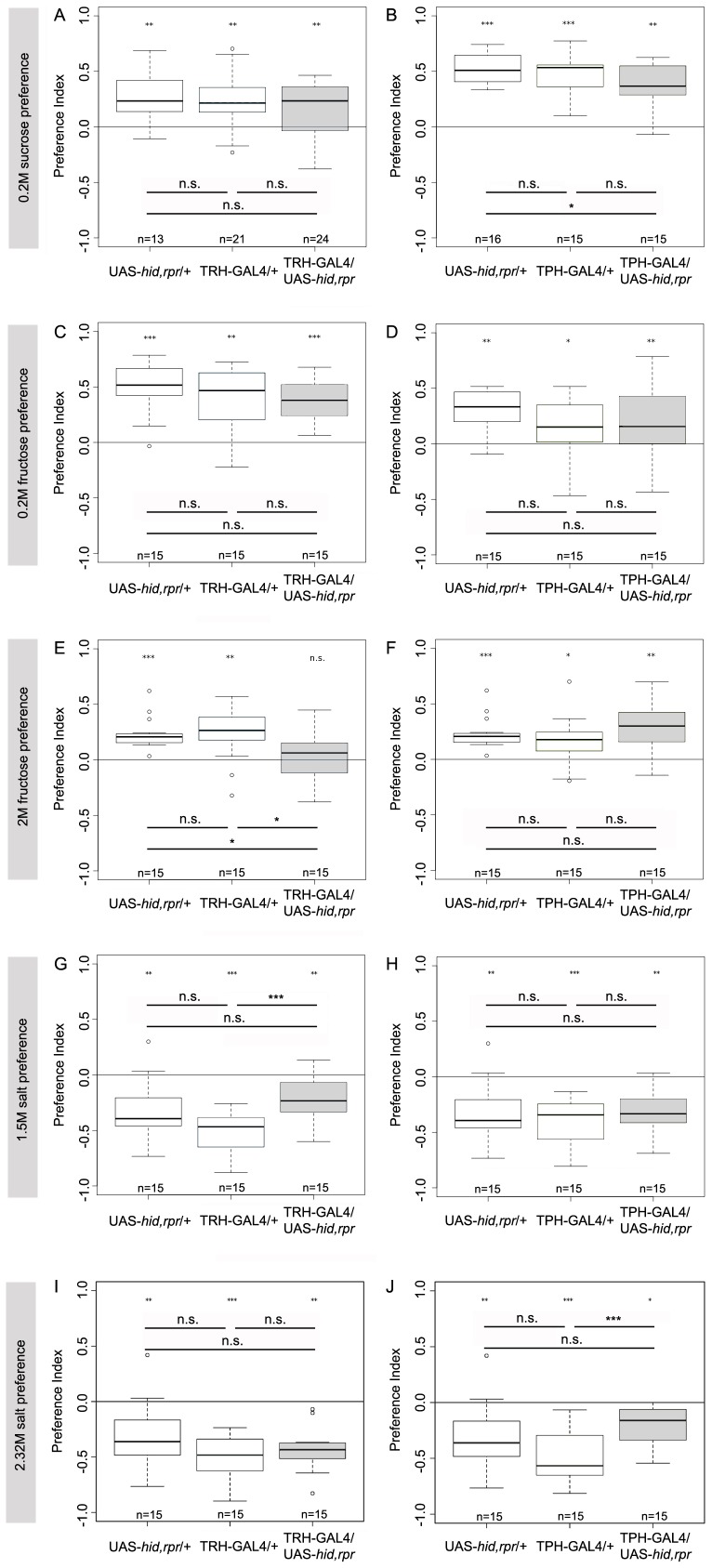
The Role of the Serotonergic System of the CNS for Gustatory Choice Behavior. Larvae were tested for their gustatory preference to different sugars (A–F) or salt (G–J) at varying concentrations. TRH-GAL4/UAS-*hid,rpr* larvae showed a strong preference for 0.2M sucrose (p<0.01) (A) and for 0.2M fructose (p<0.001) (C) with no significant difference to any control. Interestingly, TRH-GAL4/UAS-*hid-rpr* larvae did not prefer 2M fructose, whereas all controls did (p<0.05). In comparison with the control lines, no significant difference was found, except for 0.2M sucrose, where experimental larvae showed a slightly decreased preference compared to UAS-*hid,rpr*/+ (p<0.05) (B). TPH-GAL4/UAS-*hid,rpr* animals strongly preferred (p<0.01) 0.2M sucrose (B), 0.2M fructose (D) and 2M fructose (F). (E). Concerning 1.5M and 2.32M sodium chloride, we noticed a strong avoidance for both TRH-GAL4/UAS-*hid,rpr* ((G) p<0.01, (I) p<0.01) and TPH-GAL4/UAS-*hid,rpr* ((H) p<0.01, (J) p<0.05) experimental groups. The performance indices of TRH-GAL4/UAS-*hid,rpr* at 1.5M salt (G) and of TPH-GAL4-UAS-*hid,rpr* at 2.32M salt (J) were slightly reduced compared to the corresponding GAL4 control lines. Under each boxplot of the figure for each genotype the sample size is shown; n = 13−24. Asterisks above each boxplot indicate, if the data is significantly different from zero. n.s.>0.05; *<0.05; **<0.01; ***<0.001.

When preference for 0.2M sucrose was tested, TRH-GAL4/UAS-*hid,rpr* ablated larvae did not show any difference in the preference index compared to either TRH-GAL4/+ or UAS-*hid,rpr*/+ control groups (Figure 15A; p = 0.65 and p = 0.21). TPH-GAL4/UAS-*hid,rpr* larvae showed a preference that was not significantly different from TPH-GAL4/+ control larvae (Figure 15B; p = 0.15) but significantly reduced compared to UAS-*hid,rpr*/+ controls (Figure 15B; p = 0.02).

When larvae were tested for their gustatory preference toward 0.2M fructose, TRH-GAL4/UAS-*hid,rpr* ablated larvae did not show a behavioural change compared to either TRH-GAL4/+ or UAS-*hid,rpr*/+ control groups (Figure 15C; p = 0.58 and p = 0.10). A similar result was obtained for TPH-GAL4/UAS-*hid,rpr* larvae that did not perform significantly different than TPH-GAL4/+ and UAS-*hid,rpr*/+ controls (Figure 15D; p = 0.63 and p = 0.45).

However, a fructose preference test using a higher concentration (2M) revealed a difference for TRH-GAL4/UAS-*hid,rpr* larvae, in detail, they performed significantly different compared to both TRH-GAL4/+ and UAS-*hid,rpr*/+ controls (Figure 15E; p = 0.02 and p = 0.01). TRH-GAL4/UAS-*hid,rpr* larvae distributed even randomly in the plate as their preference was not significantly different from zero (p = 0.64). In contrast, the performance of TPH-GAL4/UAS-*hid,rpr* larvae was not different from the performance of TPH-GAL4/+ and UAS-*hid,rpr*/+ controls (Figure 15F; p = 0.12 and p = 0.32).

In addition to appetitive gustatory stimuli we also tested for an aversive gustatory preference applying 1.5M and 2.32M sodium chloride. When testing TRH-GAL4/UAS-*hid,rpr* ablated larvae with 1.5M sodium chloride, preference scores were significantly different from those of TRH-GAL4/+ controls, but were similar to those of UAS-*hid,rpr*/+ controls (Figure 15G; p = 0.0004 and p = 0.13). TPH-GAL4/UAS-*hid,rpr* larvae showed the same avoidance as the corresponding TPH-GAL4/+ and UAS-*hid,rpr*/+ controls (Figure 15H; p = 0.30 and p = 0.56).

For 2.32M sodium chloride, TRH-GAL4/UAS-*hid,rpr* larvae showed the same preference as both TRH-GAL4/+ and UAS-*hid,rpr*/+ controls (Figure 15I; p = 0.74 and p = 0.21). In contrast, TPH-GAL4/UAS-*hid,rpr* larvae showed a significantly reduced avoidance compared to TPH-GAL4/+ controls (Figure 15J; p = 0.0008); this was not the case when comparing ablated larvae with UAS-*hid,rpr*/+ controls (Figure 15J; p = 0.10). Taken together, when testing a set of appetitive and aversive taste stimuli at different concentrations larvae lacking almost all 5HT neurons in the CNS in some cases behaved significantly different than controls. Thus, we cannot exclude that 5HT signalling is necessary for particular aspects of gustation. Nevertheless, because in none of the cases we got a clear phenotype for both GAL4 lines, 5HT may not be necessary for basic larval orientation based on gustatory cues.

### TRH-GAL4 and TPH-GAL4 Neurons are not Necessary for Overall Phototaxis

**Figure 16:**
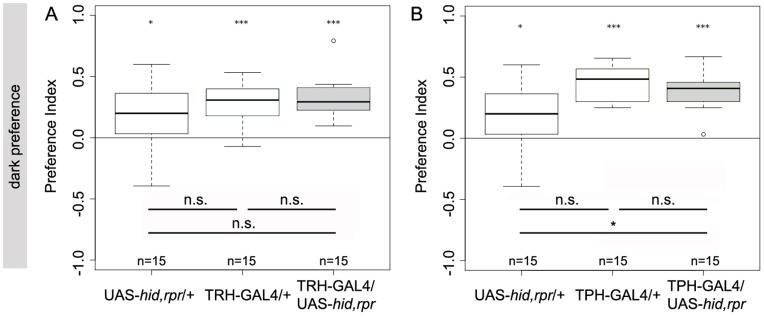
The Serotonergic Neurons of the CNS are not Necessary for Phototaxis. Preference for darkness was tested for TRH-GAL4/UAS-*hid,rpr* and TPH-GAL4/UAS-*hid,rpr* larvae as well as for driver and effector line controls. (A) TRH-GAL4/UAS-*hid,rpr* larvae showed a strong preference for light (p<0.001) and did not show any significant difference to UAS-hid,rpr/+ nor to TRH-GAL4/+. Also, TPH-GAL4/UAS-*hid,rpr* larvae preferred darkness (p<0.001), whereas TRH-GAL4/UAS-*hid,rpr* did not show any difference to the controls (A), TPH-GAL4/UAS-*hid,rpr* had a slightly higher preference compared to UAS-*hid,rpr* controls (B). Under each boxplot of the figure for each genotype the sample size is shown; n = 15. Asterisks above each boxplot indicate, if the data is significantly different from zero. *<0.05; ***<0.001.

To analyse if 5HT is required for larval phototaxis, we tested if feeding third instar larvae that lack most of the 5HT system within the CNS (Figure 5) prefer darkness against light as they do under normal conditions [13]. To this end we put 30 larvae onto a neutral agar plate and let them choose for 5 minutes between two illuminated and two dark quadrants [13], [71]. In this situation, TRH-GAL4/UAS-*hid,rpr* ablated larvae did not perform significantly different form either TRH-GAL4/+ or UAS-*hid,rpr*/+ control larvae (Figure 16A; p = 0.77 and p = 0.14, respectively). Similarly, TPH-GAL/UAS-*hid,rpr* larvae were not significantly different compared to TPH-GAL4/+ (p = 0.45) and they performed even slightly better than UAS-*hid,rpr*/+ control larvae (Figure 16B; p = 0.02). Therefore the larval 5HT neurons appear dispensable for larval phototaxis under our test conditions.

### The Role of TRH-GAL4 and TPH-GAL4 Positive Neurons in Appetitive Olfactory Learning

**Figure 17:**
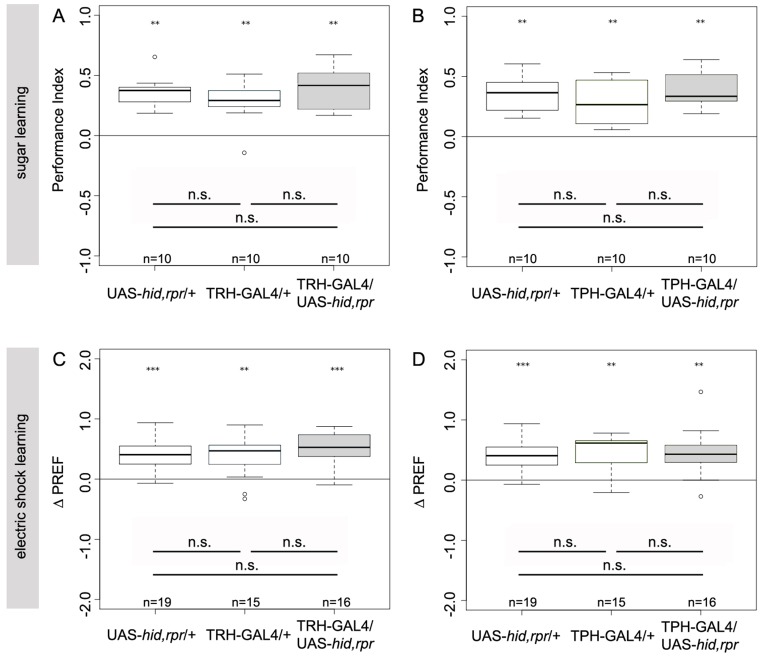
The Serotonergic Neurons of the CNS are not Necessary for Appetitive and Aversive Olfactory Learning. For testing appetitive olfactory learning, we utilized a two-group, reciprocal training design consisting of two half trials that give rise to a final performance index. Third instar larvae lacking serotonergic neurons preferred an odor that was paired with 2-M fructose (A, B). Using a single odor, non-reciprocal standard assay for aversive odor-shock learning third instar larvae lacking serotonergic neurons avoided the odor paired with pulses of electric shock (C, D). In both learning experiments, TRH-GAL4/UAS-*hid,rpr* larvae achieved relatively high performance scores (A) (p<0.01) (C) (p<0.001). Similar results were obtained for TPH-GAL4/UAS-*hid,rpr* larvae, which showed significant sugar learning (p<0.01) and electric shock learning (p<0.01). In none of the learning assays significant differences between experimental and control larvae were found. Under each boxplot of the figure for each genotype the sample size is shown; n = 10−16. Asterisks above each boxplot indicate, if the data is significantly different from zero. **<0.01; ***<0.001.

For testing appetitive olfactory learning as described in earlier studies, we utilized a two-group, reciprocal training design consisting of two half trials that give rise to a final performance index [reviewed in [24]. To interfere with 5HT neurotransmission, we again induced apoptosis by expressing UAS-*hid,rpr* via TRH-GAL4 and TPH-GAL4. After odor-sugar conditioning, TRH-GAL4/UAS-*hid,rpr* larvae showed the same performance as the GAL4/+ and UAS/+ controls (Figure 17A; p = 0.53 compared to TRH-GAL4/+ and p = 0.16 compared to UAS-*hid,rpr*/+). Similar results were obtained for odor-sugar learning in TPH-GAL4/UAS-*hid,rpr* larvae and the corresponding GAL4/+ and UAS/+ controls (Figure 17B; p = 0.22 compared to TPH-GAL4/+ and p = 0.80 compared to UAS-*hid,rpr*/+). Therefore we conclude that the serotonergic neurons of the CNS are not required for appetitive olfactory learning under our experimental conditions.

### The Role of TRH-GAL4 and TPH-GAL4 Positive Neurons in Aversive Olfactory Learning

For this experiment we utilized a nonreciprocal training design as recently established [28]. After olfactory conditioning using electric shock as an aversive US, TRH-GAL4/UAS-*hid,rpr* ablated larvae showed the same performance as the GAL4/+ and UAS/+ control groups (Figure 17C; p = 0.24 compared to TRH-GAL4/+ and p = 0.16 compared to UAS-*hid,rpr*/+) and a similar result was obtained after odor-electric shock training of TPH-GAL4/UAS-*hid,rpr* larvae and the corresponding GAL4/+ and UAS/+ controls, i.e., there was no significant difference in ΔPREF detectable (Figure 17D; p = 0.37 compared to TPH-GAL4/+ and p = 0.70 compared to UAS-*hid,rpr*/+). Therefore, the serotonergic neurons in the larval CNS are not necessary for aversive olfactory learning under our experimental conditions.

## Discussion

### The Serotonergic System during *Drosophila* Development

Serotonin is an indolamine which acts as a neurotransmitter or neuromodulator in the CNS in the majority of animal phyla [42]. Studies on its distribution in the CNS of several insect species have been made possible by the availability of specific antibodies against 5HT [41], [72]. In *Drosophila,* Lundell and Hirsh (1994) studied the differentiation of larval 5HT neurons by investigating the onset of the 5HT- and Dopa decarboxylase-immunoreactivity (DDC-IRy) in staged embryos. 5HT-IRy first appears at stages 16–17 shortly after the emergence of DDC-IRy in these cells. Staining has been initially detected in both neuronal processes and cell bodies. Lundell et al. (1996) provided evidence that the two 5HT cells in each hemineuromere of the VNC are part of the same small cell lineage of the neuroblast NB7–3 [73], [74]. This has also been proposed for the homologous serotonin cells of the grasshopper [75].

In larvae, 5HT neurons are predominantly bilaterally symmetrical interneurons with intrasegmental arborizations. Vallés and White (1988) previously reported that the 5HT-IR pattern consists of 84 neurons, distributed in clusters of one to five neurons each. Here, we were able to identify a similar set of about 84 neurons plus a small number of additional cells (Table1; Figure 1). In total we identified about 96 5HT neurons in the third instar larval brain. Vallés and White (1988) classified the 5HT neurons of the two brain hemispheres into SP1, SP2, LP1 and IP clusters consisting of three, four, three to four and two neurons per hemisphere, respectively. We were able to confirm these results (Table1; Figure 1), but we suggest that there might be only a single neuron for the SP1 cluster. However, this was also originally reported as two of the three neurons in the SP1 cluster emerge only during metamorphosis [50]. For the SOG, thoracic and abdominal ganglia, Vallés and White (1998) described a segmental pattern of 14 bilaterally symmetrical clusters of 5HT-positive somata, i.e., three for the suboesophageal ganglion (SE1, SE2, SE3), three for the thoracic ganglion (T1, T2, T3), and eight (A1–A8/A9). In general, they counted two 5HT positive cells per cluster except for SE2, SE3, and T1, which comprise three cells and A8/A9 including one cell. Here we confirmed the presence of all of these neurons, but in addition repeatedly visualized two to four 5HT neurons per side at the anterior tip of the SOG, a new cluster which we termed SE0 (Table1; Figures 1, 2 and 3). Moreover, for the SE2 and SE3 clusters we counted about five instead of three neurons per hemineuromere. The cell bodies of these two extra cells were located posteriolateral within one hemineuromere, which is clearly distinct from the other three anteriomedial somata (Table1; Figures 2 and 3). For each of the thoracic and abdominal neuromeres, we also counted about two 5HT-positive cells, except for T1 with generally three and A8/A9 with one cell.

Thus we were able to identify all serotonergic neurons described in earlier studies [41], [50], [52]. However, we also repeatedly found a small set of additional cells within the SOG that were not described before. The difference very likely arises from the technical improvements that today allow the reliable detection of extremely low fluorescence levels.

Studying the metamorphosis of the serotonergic system has revealed that in general the organization of the 5HT cell clusters persists to adulthood and that they essentially occupy similar positions in the CNS as in the larval stage [50]. Only two new clusters, LP2A and LP2B, are added to the pattern during early pupal stage; they are located in the brain near the medulla neuropil [50]. In addition, as mentioned above, the SP1 cluster is enlarged by increasing the cell number from one to three. Thus, the basic organization in terms of cell number is almost identical between the larval and adult stage [50]. However, new adult-specific structures established during metamorphosis like the central complex and the optic lobes must be innervated, which suggests a massive reorganization of axonal processes, terminals and dendritic arbors of the larval 5HT neurons.

### Single-Cell Analysis of the Serotonergic System using a Triple Staining Protocol

In their single-cell analyses, Chen and Condron (2008) characterized serotonergic cells of the VNC, while Roy et al. (2007) described a pair of contralaterally projecting serotonin-immunoreactive deutocerebral (CSD) interneurons of the IP cluster. For visualizing single serotonergic neurons, both groups used two sets of antibodies, anti-GFP and anti-5HT. However, the lack of neuropil staining in this method renders cellular localization difficult. We used a triple staining protocol using anti-5HT, anti-CD8 (instead of anti-GFP) and the neuropil markers anti-FasciclinII (FasII)/anti-Cholineacetyltransferase (ChAT). Our protocol enables us to visualize and examine single cells in great detail and to precisely locate them in their particular CNS region. For nearly all 5HT cells we were able to reveal their detailed anatomy, except for those innervating the LON and those of the SE1 neuromere. In general every 5HT cell has its symmetrical counterpart on the contralateral side. Most of the 5HT neurons stay with their arborizations within the same neuromere and look like interneurons. Moreover, 5HT neurons of subsequent neuromeres often resemble each other, which is particularly obvious for thoracic and abdominal neuromeres. Interestingly we did not find any cell that innervates the larval MBs, which is different compared to the adult stage. Here, the dorsal paired medial neurons innervate intensively the mushroom bodies and were recently shown to be serotonerigc. A comprehensive set of studies exists that shows that serotonergic neurons, DPM neurons and also 5HT receptors are involved in adult olfactory learning [76] [77] [78] [79] [80] [81] [82]. However at the larval stage 5HT neurons of the CNS seem to be not necessary for olfactory learning based on the behavioral and anatomical data of our study. However, we did not distinguish between nutrition independent and nutrition dependent appetitive olfactory learning. As it was recently shown that fructose offers at least these two types of appetitive reinforcement, it would be necessary to retest, if serotonin is only involved in one of these reward systems [83].

### The Role of the Serotonergic System for Larval Behavior

Several groups proposed independently the involvement of serotonin in *Drosophila* larval behavior [42], [59], [60]. The argument for this idea was initially a circumstantial one, based on the anatomical organization of the larval 5HT system. In particular, its innervation of the pharyngeal muscles, the proventriculus and the midgut implied a possible function in larval feeding [50], [52]. The same studies also identified 5HT neurons innervating the ring gland, i.e., the major larval endocrine organ, which suggested a role of 5HT in regulating larval neuroendocrine activity [50], [52]. Following the same logic, another study identified a single 5HT neuron arborizing in the ALs and adjacent parts of the deutocerebrum [53]. Based on the anatomical similarity to large-field neurons in a variety of insects [55] [56] [57] [58], these reports suggested that these neurons might be triggered by mechanosensory stimulation to release serotonin for modulating the threshold of odorant detection.

More recent studies addressed the role of 5HT in larval behavior directly by means of genetic intervention [42], [59], [60]. Moncalvo and Campos (2009) suggested that the activity of serotonergic neurons contribute to the control of light-induced larval locomotion. Using a so called ON/OFF assay, they showed that Ddc-GAL4;UAS-TNT-G larvae which are not able to release neurotransmitters from the 5HT Ddc-GAL4-positive neurons, pause more and longer in the presence of light. The behavioural phenotype was more pronounced for wandering than for feeding third instar larvae. As Ddc-GAL4 is expressed in dopamine-, serotonin- and corazonin-positive cells, they further restricted the expression pattern to 5HT neurons, by using the same TPH-GAL line as we did (called TRH in their study; tryptophan hydroxylase). Interestingly, expression of UAS-TNT via TPH-GAL4, for blocking neurotransmission in 5HT-positive cells, led to a similarly significant change in the response to light. Moreover, the same behavioural change also appeared in a TRH mutant, and a reduced response to light was seen when overexpressing the 5HT1A receptor pan-neuronal [59]. Thus, it was concluded that 5HT modulates visually guided behaviour. At first sight, our results which show that the naïve dark preference after five minutes does not depend on the 5HT system might contradict these findings (Figure 15). However, we tested for phototaxis with a temporal resolution of five minutes which is hard to compare with the specifically regulated ON/OFF response to light stimuli within less than a second. Thus it remains possible that 5HT is not required for the general orientation of the larvae in a constant light-dark environment but may adjust the fine tuning of the visual response to light onset.

Neckameyer and colleagues described the existence of two different enzymes that hydroxylate trypthophan, the first step in serotonin synthesis [42], [84]. They found that one of them is also non-neuronal (called TPH1 or DTPHu) while the other is neuron-specific (called TPH2 or DTRHn, the subject of this study) [42]. Using a null mutation for DTRHn, they comprehensively described the behavioural relevance of 5HT for *Drosophila* at different developmental stages. For larvae they found that the DTRHn null mutant is significantly impaired in feeding due to a reduced number of mouthhook contractions [42]. Furthermore, olfactory perception was modified for one of two odors tested: naïve wildtype larvae usually do not show any preference for nonanol, while DTRHn mutants avoided the odor [42]. For the second odor, heptanol, there was no difference for the olfactory preference. Larval locomotion (number of body wall contractions per minute) was unaltered in the DTRHn null mutant.

As we did not test larval feeding, we are unable to speculate on a possible role of the 5HT system in this behaviour. However, Neckameyer and colleagues also showed that increased 5HT levels reduce feeding whereas reduced 5HT levels increase appetite [60]. Thus it was suggested, similar to most species tested so far, that feeding in *Drosophila* larvae seems to be regulated by 5HT.

We did not detect any obvious locomotion phenotype in our 5HT ablation assays by expressing *hid* and *rpr* in nearly all serotonergic neurons (however we have not tested it by using recently established tracking setups). Moreover, for AM and BA we saw no change in the naïve olfactory preference (Figure 14). Thus, serotonergic neurons in the CNS might be involved in the sensory processing of specific odors, like nonanol, but not other of other odors like amylacetate, BA and heptanol [42]. However, we cannot exclude the possibility that 5HT is necessary for larval olfactory sensation as the 5HT-positive CSD neurons which innervate the AL were not always completely ablated in our approach (Figure 5). Thus, 5HT modulation in surviving CSD neurons may be sufficient for innate odor processing.

In addition, we want to mention that our analysis is restricted to the functional analysis of 5HT positive neurons by ablating nearly all of these neurons only within the larval CNS. Therefore, it is still possible that 5HT regulates each of the described behaviours, if i) remaining 5HT cells outside of the larval CNS control these behaviors; ii) compensatory mechanisms during larval development exist that take over behavioural functions; iii) antagonistic sets of 5HT neurons exist that inhibit and activate a particular behaviour. Here, ablation of both sets would not change the net output. iv) It was reported that 5HT neurons signal onto at least four different types of 5HT receptors, called 5HT1A, 5HT1B, 5HT2 and 5HT7. Although, all of them are G-protein coupled receptors, 5HT1A and 5HT1B inhibit adenylate cyclase, whereas 5HT7 stimulates it [77], . Thus, if postsynaptic cells antagonistically regulate larval behaviors also the deletion of their input would not change the net output of the system. And indeed, based on promoter GAL4 expression studies it is possible that 5HT1A, 5HT1B and 5HT7 receptor cells may receive serotonergic input in the protocerebrum, SOG, thoracic and abdominal ganglion; whereas 5HT2 receptor expression seems to be restricted to glia cells at the third instar stage [77], .

### Global Role of the Serotonergic System

In adult *Drosophila* and other insects, 5HT has been reported to modulate circadian rhythms, reproduction, feeding, heart rate and locomotion [90] [91] [92] [93] [94], besides light-dependent locomotion, olfaction and feeding [42], [59], [60]. However, flies having diminished neuronal 5HT are still viable and fertile. This suggests that serotonin is either also processed cells outside of the CNS, or it may only modulate many behaviors, but is not the principal neurotransmitter for any of these. The second idea is somehow supported by our various behavioral assays with larvae lacking most of the 5HT neurons (Figures 14, 15, 16 and 17). Neither olfactory and light, nor gross gustatory perception was disabled, and even olfactory associative learning was unaffected by dramatically reduced 5HT signaling. Interestingly, in mammals 5HT modulates appetite, sleep, learning and memory, temperature regulation, cognition, sensory processing, motor activity and sexual behavior, as well as emotional behaviors including anxiety and aggression [95] [96] [97] [98]. Thus, in mammals, too, 5HT orchestrates the neuronal network for a comprehensive set of behavioral functions, but is not required *per se* for distinct behaviors. Given the conserved functional role of 5HT between mammals and insects, it would now be interesting to analyze in more detail how behavioral responses are finely tuned by 5HT in *Drosophila* larvae. The underlying neuronal and molecular mechanisms might then be valid not only for insects but for mammals as well.

### Outlook

Our comprehensive analysis of the larval 5HT system describes its basic anatomy and provides insights into the relevance of the system for larval behavior. Given the surprising observation that 5HT in the larval CNS is not required for innate behavioral responses triggered by visual, olfactory, only partially by gustatory cues and does not seem to be implicated in olfactory associative learning, one can now address the question if 5HT is involved in the fine tuning of these behaviors (rather than their implementation). Also a set of genetic tools for interfering with specific parts of the 5HT molecular pathway is emerging [42], [59], [60], [88−90], . By that developmental as well as antagonistic function of individual enzymes, receptors and neurons can be revealed, even outside of the larval CNS. In addition, sophisticated assays exist for behavioral tracking with high temporal resolution and automated data analysis [102], [103]. Thus, we can now address in more detail how 5HT provides larvae with a variety of behavioral outputs, in order to adapt environmental and developmental changes by adjusting multifunctional neuronal circuits.

## Materials and Methods

### Fly Strains

Fly strains were reared on standard *Drosophila* medium at 25°C or 18°C with a 14/10h light/dark cycle, or in constant darkness in case of the hsp70-flp;TRH-GAL4/+;UAS>CD2y^+^>mCD8::GFP/+ larvae or hsp70-flp;TPH-GAL4/+;UAS>CD2y^+^>mCD8::GFP/+ larvae [63]. TPH-GAL4 was provided by Jongkyeong Chung and TRH-GAL4 [39] by Serge Birman [40]. Construction of this diver will be described elsewhere. For the behavioral experiments, UAS-*hid,rpr* effectors inserted on the X chromosome were used to ablate serotonergic neurons, by crossing to the GAL4-driver lines TRH-GAL4 or TPH-GAL4 [36[ [37[ [38[ [39[ [40]. Heterozygous controls were obtained by crossing GAL4-driver and UAS-effector to *w^1118^*. For visualizing neurons, we crossed TRH-GAL4 and TPH-GAL4 with UAS-*mCD8::GFP*. The pre- and postsynaptic regions of the TRH-GAL4 and TPH-GAL4 expressing neurons were visualized using UAS-*Dscam[17.1]::GFP* or UAS-*n-syb::GFP* [66] [67] [68]. For single-cell staining, *y w hsp70-flp; Sp/CyO; UAS>CD2y^+^>mCD8::GFP/TM6b* virgins were crossed to TRH-GAL4 or TPH-GAL4 males. A single heat shock at 37°C for 18 min was applied by placing the vials in a water bath. For the onset of heat shock, larvae of different ages ranging from 0 to 200 hours after egg laying were chosen [19].

### Immunofluorescence Antibodies

To analyze the expression pattern of TRH-GAL4 and TPH-GAL4, we used a rat CD8 antibody (anti-CD8; Molecular Probes, Eugene, OR, 1∶200), a rabbit anti-serotonin antibody (anti-5HT, Sigma, 1∶500) and two different mouse antibodies for staining the neuropils (ChAT4B1; DSHB, Iowa City, IA, 1∶150) and the axonal tracts (1d4 anti-Fasciclin II; DSHB, Iowa City, IA; 1∶50), respectively. The same set of primary antibodies was also used for the single-cell approach. In the experiments aimed at visualizing pre- and postsynaptic structures of TRH-GAL4 and TPH-GAL4, we used rabbit anti-GFP (GFP, Molecular Probes, 1∶1000) and the two mouse antibodies mentioned above for staining the neuropil (ChATB1; DSHB, Iowa City, IA, 1∶150) and the axonal tracts (1d4 anti-Fasciclin II; DSHB, Iowa City, IA, 1∶50), respectively. Goat anti-rat IgG Alexa Fluor 488 (Molecular Probes, 1∶200), goat anti-rabbit IgG Alexa Fluor 568 (Molecular Probes, 1∶200) and goat anti-mouse IgG Alexa Fluor 647 (Molecular Probes, 1∶200) were used as secondary antibodies.

### Immunostaining

Third instar larvae were put on ice and dissected in phosphate-buffered saline (PBS). Brains were fixed in 3.6% formaldehyde (Merck, Darmstadt) in PBS for 25 min. After four times rinsing in PBT (PBS with 3% Triton-X 100, Sigma-Aldrich, St. Louis, MO), brains were blocked with 5% normal goat serum (Vector Laboratories, Burlingame, CA) in PBT for 1.5 hours and then incubated for two days with primary antibodies at 4°C. Before applying the secondary antibodies for one day at 4°C, brains were washed six times with PBT. Finally, brains were washed five times with PBT and once with PBS, mounted in Vectashield (Vector Laboratories) between two cover slips and stored at 4°C in darkness. Images were taken with a LeicaTCS SP5 confocal microscope with x20 or x63 glycerol objectives. The resulting image stacks were projected and analyzed with Image-J (NIH) software. Contrast and brightness adjustment as well as rotation and organization of images were performed in Photoshop (Adobe Systems Inc., San Jose, CA).

### Behavioral Experiments

For all behavioral assays, flies were allowed to lay eggs for two days. Experiments were performed at the fifth or sixth day after egg laying. Third instar larvae used for the behavioral experiments were therefore 96−144 hours old; only feeding stage larvae were taken. For all experiments, groups of about 30 larvae were used.

### Olfactory, Gustatory and Visual Preference Tests

For olfactory and visual preference tests, 2.5% agarose solution (Sigma Aldrich) was boiled in a microwave oven and filled as a thin layer into Petri dishes (85mm diameter). After cooling, closed Petri dishes were kept at room temperature and were used on the same day or on the next day. For gustatory preference tests, the procedure was the same, except that after cooling, the agarose was removed from half of the plate. The empty half was filled by 2.5% agarose solution containing either 0.2M sucrose, 0.2M fructose, 2M fructose, 1.5M or 2.32M sodium chloride. These gustatory test dishes were used on the same day.

For olfactory preference assays, 10 µl of either pure benzaldehyde or diluted amylacetate (1∶250) were loaded into a Teflon container [24]. Olfactory preferences were tested by placing 30 larvae in the middle of the Petri dish that contained an odor containing Teflon container on one side and an empty container on the other side. Larvae were then counted after 5 minutes on the odor, non-odor and neutral side (an area of about 1 cm diameter running vertically in the middle of the plate). For gustatory preference tests, 30 larvae were put in the middle of a Petri dish that contained pure agarose on one side and agarose plus a gustatory stimulus (sucrose, fructose or salt) on the other side. Larvae were counted after 5 minutes on the odor, non-odor and neutral side (an area of about 1 cm diameter running vertically in the middle of the plate). For dark preference tests, 30 larvae were placed in the middle of a pure agarose plate in which alternating quarters were illuminated by light (about 800 lux) and dark. Larvae in illuminated and dark quarters were counted after 5 minutes.

In all preference tests, a preference index was calculated:



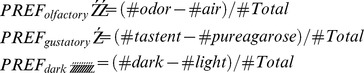



### Appetitive Olfactory Learning

To test for larval appetitive olfactory learning, a reciprocal design was applied consisting of two half trials that finally lead to the calculation of a performance index. In detail, petri dishes with 2.5% agarose (prepared as described above) and others with 2M fructose diluted in 2.5% agarose were used for testing appetitive olfactory learning. 30 third instar larvae (96−144 hours old) were put on the midline of the pure agarose Petri dish containing two Teflon containers. The containers were loaded with 10 µl diluted amylacetate (AM) (1∶250 in paraffin oil) acting as a non-reinforced odor. After five minutes the larvae were transformed to a sugar plate containing two Teflon containers filled with 10 µl benzaldehyde (BA) (reinforced odor). Five minutes later the larvae were transferred to a new pure agarose dish with AM odor as a cue starting a new training cycle. Immediately after three training cycles, the larvae were tested for odor preference during five minutes on a pure agarose plate with both BA and AM Teflon containers on opposite sides of the dish. In the end of the test, the larvae were counted at each side of the plate and a preference value for BA (PREF_AM/BA+_) was calculated (see below). Another group of larvae was tested reciprocally, i.e., the sugar plates were combined with AM (CS+) and the pure agarose plates with BA (CS-), which also allows calculation of a preference value for AM (PREF_AM+/BA_). The final preference index (PI) was determined by dividing the difference of the two preference values (PREF_AM+/BA_ – PREF_AM/BA+_)) by two. All assays were performed under the fume hood with normal light at 21°C.
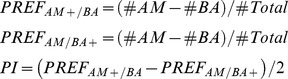



### Aversive Olfactory Learning

For investigating aversive olfactory learning, we used pure 2.5% agarose Petri dishes. In contrast to appetitive olfactory learning assays, we used electric shock as a negative stimulus instead of fructose as positive reinforcer. About 30 larvae had to undergo a pretest to assess their naïve BA preference. During training, larvae were exposed to BA for one minute, which was paired for the last 30 sec by a 100 V AC electric shock. This was followed by a five minutes resting phase on a pure agarose plate. The training was repeated five times. Immediately after training, larvae were tested for five minutes for their BA preference. A ΔPREF index was calculated by subtracting the BA preference after training from the naïve BA preference before training. For more details see also [28].
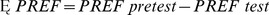



### Statistical Methods

For the comparison between genotypes, Wilcoxon rank sum test was used. To compare single genotypes against chance level, we used the Wilcoxon signed ranked test. All statistical analyses and visualizations were done with R version 2.8.0. Figure alignments were done with Adobe Photoshop. Data were presented as box plots, including all values of a given genotype, 50% of the values being located within the box. The median performance index was indicated as a bold line within the box plot. Significance levels between genotypes shown in the figures refer to the p-value obtained in the statistical tests.
